# Mechanism of action of DSP-7888 (adegramotide/nelatimotide) Emulsion, a peptide-based therapeutic cancer vaccine with the potential to turn up the heat on non-immunoreactive tumors

**DOI:** 10.1007/s12094-022-02946-0

**Published:** 2022-09-23

**Authors:** Natsuko Suginobe, Megumi Nakamura, Yosuke Takanashi, Hitoshi Ban, Masashi Gotoh

**Affiliations:** Sumitomo Pharma Co., Ltd., 3-1-98, Kasugade Naka, Konohana-ku, Osaka, 554-0022 Japan

**Keywords:** DSP-7888, Adegramotide, Nelatimotide, Therapeutic cancer vaccine, Wilms' tumor 1 (WT1), Immune checkpoint inhibitor, Mechanism of action, Tumor microenvironment, Cytotoxic T lymphocytes, Helper T lymphocytes

## Abstract

**Background:**

Wilms’ tumor 1 (WT1) is highly expressed in various solid tumors and hematologic malignancies. DSP-7888 (adegramotide/nelatimotide) Emulsion is an investigational therapeutic cancer vaccine comprising three synthetic epitopes derived from WT1. We evaluated the mechanism of action of DSP-7888 Emulsion, which is hypothesized to induce WT1-specific cytotoxic T lymphocytes (CTLs) and helper T lymphocytes (HTLs).

**Methods:**

The ability of nelatimotide and adegramotide to induce WT1-specific CD8^+^ T cells and CD4^+^ T cells was assessed in human peripheral blood mononuclear cells (PBMCs). The ability of DSP-7888 Emulsion to induce WT1-specific CTLs in vivo was assessed using human leukocyte antigen-I (HLA-I) transgenic mice. To assess how adegramotide, the helper peptide in DSP-7888 Emulsion, enhances WT1-specific CTLs, HLA-I transgenic mice were administered DSP-7888 or nelatimotide-only Emulsion. Interferon-gamma secretion under antigen stimulation by splenocytes co-cultured with or without tumor cells was then quantified. The effects of combination treatment with DSP-7888 Emulsion and an anti–programmed cell death protein 1 (PD-1) antibody on tumor volume and the frequency of tumor-infiltrating WT1-specific T cells were assessed in HLA-I transgenic mice implanted with WT1 antigen-positive tumors.

**Results:**

The peptides in DSP-7888 Emulsion were shown to induce WT1-specific CTLs and HTLs in both human PBMCs and HLA-I transgenic mice. Unlike splenocytes from nelatimotide-only Emulsion-treated mice, splenocytes from DSP-7888 Emulsion-treated mice exhibited high levels of interferon-gamma secretion, including when co-cultured with tumor cells; interferon-gamma secretion was further enhanced by concomitant treatment with anti-PD-1. HLA-I transgenic mice administered DSP-7888 Emulsion plus anti-PD-1 experienced significantly greater reductions in tumor size than mice treated with either agent alone. This reduction in tumor volume was accompanied by increased numbers of tumor-infiltrating WT1-specific CTLs.

**Conclusions:**

DSP-7888 Emulsion can promote both cytotoxic and helper T-cell-mediated immune responses against WT1-positive tumors. Adegramotide enhances CTL numbers, and the CTLs induced by treatment with both nelatimotide and adegramotide are capable of functioning within the immunosuppressive tumor microenvironment. The ability of anti-PD-1 to enhance the antitumor activity of DSP-7888 Emulsion in mice implanted with WT1-positive tumors suggests the potential for synergy.

## Background

*Wilms’ tumor 1* (*WT1*) is overexpressed in a variety of solid tumor types and hematologic malignancies [1, 2]. For example, more than 90% of serous ovarian tumors are positive for WT1, and its presence has been shown to be specific enough to distinguish serous ovarian tumors from those of other cellular origin [3, 4]. In addition, while functionally absent in normal brain tissue, WT1 levels are highly elevated in patients with glioblastoma multiforme (GBM), making it a promising immunotherapeutic target [1, 5, 6]. Under normal physiologic conditions, WT1 plays a pivotal role in the development and homeostasis of several organs, including the kidney, gonads, and heart [1, 2]. However, WT1 can also function as an oncogenic driver. By regulating endothelial cell proliferation and migration, WT1 has been implicated in tumor angiogenesis [7]. Downregulation of WT1 has been shown to decrease tumorigenicity by arresting cellular growth and proliferation, promoting apoptosis, and reducing cellular invasiveness [5, 8–13]. WT1 has been ranked as the most promising target antigen for therapeutic cancer vaccine development [14].

Most WT1 peptide-based vaccines previously tested in patients with cancer contained only human leukocyte antigen (HLA)-I epitopes and, therefore, only elicited WT1-specific cytotoxic (CD8^+^) T lymphocytes [15–21]. That these vaccines had only minimal clinical effects in patients with solid tumors may have been related to the lack of helper (CD4^+^) T-cell induction. Helper T cells play an important role in adaptive immune responses by priming and recruiting CD8^+^ T cells and activating cytotoxic T-cell memory responses [22–24]. The ability of CD4^+^ T cells to augment CD8^+^ T-cell antitumor responses is supported by data from a WT1-based therapeutic cancer vaccine containing only HLA-II antigens. This vaccine was shown to not only stimulate the proliferation of WT1-specific helper T cells, but to enhance WT1-specific cytotoxic T-cell numbers [25]. Therefore, the antitumor activity of WT1-based therapeutic cancer vaccines will likely be maximized by the inclusion of epitope sequences that stimulate both cytotoxic and helper T cells.

DSP-7888 (adegramotide/nelatimotide) Emulsion, also known as Ombipepimut-S, is an investigational therapeutic cancer vaccine composed of three synthetic epitopes derived from WT1 [26, 27]. Nelatimotide is a synthetic peptide consisting of two CD8^+^ T cell epitopes: WT1_126–134_ (RMFPNAPYL) and a modified version of WT1_235–243_ (CYTWNQMNL). In a phase I clinical trial, two patients with advanced glioblastoma who received WT2725, an earlier generation product containing only the WT1_126–134_ peptide, achieved complete response and survived for ≥ 2 years [28]. Relative to the endogenous protein, the amino acid at the second position in modified WT1_235–243_ was changed from methionine to tyrosine to enhance the binding avidity to HLA-A*24:02 [29]. Nelatimotide may be processed intracellularly by antigen-presenting cells and presented on HLA-A*02:01, HLA-A*02:06, or HLA-A*24:02. Collectively, these three HLA-I alleles are present in approximately 60% of Americans [30], 74% of those living in Japan [31], and 52 to 58% of Europeans [32–35]. The third synthetic epitope in DSP-7888 is adegramotide (WT1_34–51_: WAPVLDFAPPGASAYGSL). Within this peptide is an HLA-A*02:01-targeting sequence (VLDFAPPGA) that corresponds to amino acids 37 to 45 of the endogenous WT1 protein [36]. Thus, adegramotide can induce the activation of both cytotoxic and helper T-cell-mediated immune responses.

Immune checkpoint inhibitors, such as those targeting programmed cell death protein 1 (PD-1) or its ligand (PD-L1), have transformed the treatment of cancer. However, the efficacy of these agents is limited to “hot tumors,” which are characterized by an influx of T lymphocytes [37, 38]. PD-L1 inhibitors have been explored in patients with platinum-resistant ovarian cancer or recurrent GBM, but none has been shown in the phase III setting to extend overall survival [39, 40]. This is a possible consequence of the immunologically “cold” nature of these tumors [40, 41]. It has been hypothesized that the immunosuppressive mechanisms associated with cold tumors must be overcome before treatment with immune checkpoint inhibitors can be effective [41, 42] Thus, patients with platinum-resistant ovarian cancer or recurrent GBM are in need of novel agents, such as therapeutic cancer vaccines, with the ability to convert “cold tumors” into “hot tumors.” In this report, we describe the results of preclinical experiments that have helped to elucidate the mechanism of action of DSP-7888 Emulsion, as well as the potential to combine DSP-7888 Emulsion with an immune checkpoint inhibitor.

## Methods

### Cells

Human peripheral blood mononuclear cells (PBMCs) were purchased from Cellular Technology Ltd. EL4 cells were purchased from Dainippon Pharma, Co., Ltd.

EL4-A2402/K^b^ cells (EL4 cells stably expressing HLA-A2402/K^b^ [chimera consisting of the α1 and α2 domains of HLA-A*24:02 and the α3, transmembrane, and cytoplasmic domains of mouse H-2 Kb]) and EL4-A2402/K^b^-WT1 cells (EL4-A2402/K^b^ cells stably expressing modified WT1_235-243_ peptide) were established in our laboratory.

EL4 S3^−^ Rob cells and EL4 HHD cells (EL4 S3^−^ Rob cells stably expressing HHD [chimera consisting of the α1 and α2 domains of HLA-A*02:01 and the α3, transmembrane, and cytoplasmic domains of mouse H-2D^b^]) were established in Institut Pasteur [43].

LLC-HHD-WT1 cells (mouse Lewis lung carcinoma cells stably expressing HHD and WT1_126-134_ peptide) were established in our laboratory.

### Animals

HLA-A*02:01 transgenic mice (*HLA-A2.1/HLA-DR1–*transgenic *H-2 class I/class II–*knockout mice) were generated at Institut Pasteur [44]. HLA-A*24:02 transgenic mice (HLA-A2402/K^b^ transgenic mice) were generated at Sumitomo Pharma [45]. In each of the animal experiments, five mice per treatment group were randomized per the “randomized block design by one variable” function in SAS version 9.4 (SAS Institute Inc.) based on baseline body weight. This sample size was estimated to be necessary and sufficient to detect significant vaccine efficacy by preliminary experiments. All mouse cages were placed in the same constant environment throughout the test period in accordance with the facility’s regulations regarding animal experimentation. Although it was stipulated in advance that animals with abnormal health (and any data derived from such animals) would be removed from the analysis, no mouse was excluded from any experiment.

### In vitro induction of WT1-specific CD8^+^ T cells by peptides in DSP-7888 Emulsion

To evaluate whether the epitopes in DSP-7888 Emulsion induce WT1-specific cytotoxic T cells, human PBMCs positive for HLA-A*02:01 or HLA-A*24:02 were incubated for 11 days with 40 µg/mL nelatimotide (Bachem) plus 40 µg/mL adegramotide (Sumitomo Pharma Co., Ltd.), or 0.2% dimethylsulfoxide (DMSO). PBMCs were suspended at 5.9–6.1 × 10^5^ cells/mL in AIM-V culture medium supplemented with human serum, non-essential amino acids, and interleukin-2 and plated into 96-well U-bottom micro plates. On Day 11, the plates were prepared for fluorescence-activated single cell sorting (FACS). T-Select tetramers (Medical & Biological Laboratories Co., Ltd.) for WT1_126–134_ /HLA-A*02:01, WT1_37–45_/HLA-A*02:01, or modified WT1_235–243_/HLA-A*24:02 were then added followed by fluorescein isothiocyanate (FITC)-conjugated mouse anti-human CD8 (BD Biosciences). The number of WT1-specific CD8^+^ T cells was measured by MACSQuant Analyzer and quantified using FlowJo software version 10.4 (BD Biosciences).

### In vitro induction of WT1-specific CD4^+^ T cells by peptide in DSP-7888 Emulsion

The induction of WT1-specific helper T cells was also measured. Human PBMCs positive for HLA-DRB1*14:54/15:01, HLA-DQB1*05:03/06:03, and HLA-DPB1*04:01/04:02were incubated for 10 days with adegramotide (Bachem). On Day 10, 40 μg/mL adegramotide or 0.125% DMSO were added and incubated for 3.5 h. Following an overnight incubation with GoldiPlug (BD Biosciences), cells were stained for CD4 (FITC-conjugated mouse anti-human CD4; BD Biosciences), fixed using a Fixation/Permeabilization Kit (BD Biosciences), and stained with phycoerythrin-conjugated mouse anti-human interferon-gamma (IFN-γ) (BD Biosciences).

### In vivo induction of WT1-specific CD8^+^ T cells by DSP-7888 Emulsion

To evaluate the ability of DSP-7888 Emulsion to induce WT1-specific cytotoxic T lymphocytes in vivo, 8-week-old female HLA-A*02:01 transgenic mice were randomized to receive a single intradermal injection of either DSP-7888 Emulsion or a control Emulsion that contained no WT1 epitopes. DSP-7888 Emulsion was injected as 100 µL/head (1 and 0.75 mg/head of nelatimotide and adegramotide, respectively). Mice were euthanized by carbon dioxide inhalation 7 days posttreatment; splenocytes were harvested and diluted to 3.7 × 10^6^ cells/mL. In parallel, splenocytes from naïve mice were harvested, diluted to 7 × 10^6^ cells/mL, and stimulated with 100 μg/mL nelatimotide (Bachem) and 100 μg/mL adegramotide (Bachem) for 1 h and used as antigen-presenting cells. The antigen-presenting cells from naïve mice and splenocytes from treated mice were mixed (1:19) and incubated in 24-well plates for 5 days. These cultured cells were then used as effector cells.

EL4 HHD cells and EL4 S3^−^ Rob cells were used as target cells. In total, 5 × 10^5^ target cells were suspended in ^51^Cr (supplied as Na_2_^51^CrO_4_ [PerkinElmer, Inc.]) and incubated for 1 h. Target cells were then pulsed with 167 µg/mL WT1_126–134_ or WT1_37–45_ peptide (Life Technologies) or 0.4% DMSO for 1 h, washed twice, and diluted to 5 × 10^4^ cells/mL.

Effector cells and target cells were added to each test well of a 96-well U-bottom plate and seeded at 4 × 10^5^ and 5 × 10^3^ cells/well, respectively. To measure the spontaneous release or the maximal release of ^51^Cr from target cells, culture medium or 1% Nonidet P-40 solution was added to target cell cultures, respectively. Each sample was plated in triplicate. The plate was then incubated for 4 h. ^51^Cr release was measured using a 2470 Wizard^2™^ Automatic Gamma Counter (PerkinElmer, Inc.). Cytotoxic activity (percent-specific lysis) was calculated as [(radioactivity in each well in the ^51^Cr-release group—mean radioactivity in the spontaneous release group)/(mean radioactivity in the maximal release group—mean radioactivity in the spontaneous release group)] × 100. The calculated cytotoxic activity of each group was compared via two-sided *t* test using SAS version 9.4.

The same experiments were performed using 8-week-old female HLA-A*24:02 transgenic mice and modified WT1_235–243_ peptide (same concentration as the WT1_126–134_ and WT1_37–43_ peptides above) (Life Technologies). In these experiments, the target cells were EL4-A2402/K^b^ cells and EL4 cells.

### In vivo induction of WT1-specific cells by nelatimotide-only Emulsion or DSP-7888 Emulsion

To assess the ability of adegramotide to enhance nelatimotide-mediated induction of WT1-specific cytotoxic T cells, 8-week-old female HLA-A*02:01 transgenic mice received 2 weekly intradermal injections of either DSP-7888 Emulsion or nelatimotide-only Emulsion. The dose of nelatimotide in DSP-7888 Emulsion and nelatimotide-only emulsion was 1 mg/head. The dose of adegramotide in DSP-7888 Emulsion was 0.75 mg/head. Mice were euthanized by carbon dioxide inhalation 7 days after the second injection. Splenocytes from each mouse were transferred in triplicate to 96-well plates coated with anti-mouse IFN-γ, as provided in the Mouse IFN-γ Enzyme-Linked Immunospot (ELISPOT) Set (BD Biosciences), and seeded at 1.25 × 10^5^ cells/well. Cells were then stimulated with 10 μg/mL WT1_126–134_ peptide (Life Technologies) or 0.025% volume DMSO (Nacalai Tesque, Inc.) and incubated for 19 h. IFN-γ was detected by biotinylated anti-mouse IFN-γ and streptavidin-HRP from the Mouse IFN-γ ELISPOT Set and AEC Substrate Set (BD Biosciences). The number of spots in each well was determined using CTL-ImmunoSpot S5 Versa Analyzer (Cellular Technology, Ltd.) and quantified using the Smart Count mode in software version 5.0.3. T-cell induction in the two treatment groups was compared via two-sided *t* test using SAS version 9.4.

### In vitro effect of PD-1 inhibition on WT1-specific T cells induced by nelatimotide-only Emulsion or DSP-7888 Emulsion

To evaluate potential synergy between DSP-7888 Emulsion and PD-1 inhibition, 8-week-old female HLA-A*02:01 transgenic mice received a single intradermal injection of either DSP-7888 Emulsion (*n* = 5) or nelatimotide-only emulsion (*n* = 5). After 7 days, mice were euthanized via carbon dioxide inhalation, and splenocytes from each group were harvested and pooled. A portion of splenocytes from each group were pulsed with 100 µg/mL WT1_126-134_ peptide for 1 h, washed twice, and used as antigen-presenting cells. LLC-HHD-WT1 cells were irradiated with X-ray, stimulated for 2 days with 100 ng/mL recombinant mouse IFN-γ (R&D Systems), and used as tumor cells. Splenocytes, antigen-presenting cells, and tumor cells were co-cultured (10:1:1) in 96-well U-bottom plates (“with tumor cells” culture). Control wells received culture medium instead of tumor cells (“without tumor cells” culture). Both the “with” and “without” tumor cell cultures were incubated with anti-PD-1 (29F.1A12, BioLegend) or an isotype control antibody (rat IgG2aκ, BD Biosciences) for 3 days. Each sample was plated in triplicate. Secreted IFN-γ was measured using the Mouse IFN-γ ELISA Set (R&D Systems).

### Effect on tumor-infiltrating WT1-specific CD8^+^ lymphocytes by PD-1 inhibition

HLA-A*24:02 transgenic mice subcutaneously implanted with EL4-A2402/K^b^-WT1 cells (five per treatment group) received intradermal DSP-7888 Emulsion plus intraperitoneal anti-PD-1 (clone RMP1-14, Bio X Cell) or DSP-7888 Emulsion plus isotype control antibody (rat IgG2aκ, Bio X Cell). DSP-7888 Emulsion was administered on days 4 and 11, and anti-PD-1 or the isotype control antibody was administered on days 4, 7, and 11; on day 4, anti-PD-1 (or the isotope control antibody) was injected immediately after DSP-7888 Emulsion. On day 15, mice were euthanized via carbon dioxide inhalation and their implanted tumors resected. Tumors were dissociated with collagenase type IV (Sigma–Aldrich) and gentleMACS^™^ Dissociator (Miltenyi Biotec). Tumor-infiltrating lymphocytes were isolated by Percoll density gradient centrifugation. Cells were then incubated at 4 °C for 10 min with FACS buffer and mouse Fc receptor blocking reagent (rat anti-mouse CD16/CD32, BD Pharmingen). Cells were then stained with 3 µL modified WT1_235–243_ tetramer (T-Select HLA-A*24:02 WT1 [mutant] Tetramer-CYTWNQMNL-PE; Medical & Biological Laboratories Co., Ltd.) followed by FITC-conjugated rat anti-mouse CD8a (BD Pharmingen). Dead cells were stained with fixable viability stain 450 reagent (BD Biosciences) and removed from the analysis. The number of WT1-specific CD8^+^ T cells was measured by MACSQuant Analyzer and quantified using FlowJo software version 10.4.

### Antitumor effect of combination treatment with DSP-7888 Emulsion and PD-1 inhibition

HLA-A*24:02 transgenic mice subcutaneously implanted with EL4-A2402/K^b^-WT1 cells were treated with vehicle (water for injection emulsified with Montanide ISA™ 51 VG) plus phosphate buffered saline (PBS), DSP-7888 Emulsion plus isotype antibody, vehicle plus anti-PD-1, or DSP-7888 Emulsion plus anti-PD-1 (five mice per treatment group). Vehicle or DSP-7888 Emulsion were intradermally injected on Days 1 and 8. PBS or antibodies were intraperitoneally injected on Days 1, 4, 8, and 11. Tumor diameter was measured by a blinded technical assistant on Days 4, 7, 10, 11, 16, and 21. Tumor volume was calculated as (major axis × minor axis × minor axis)/2. Tumor volume was compared between each treatment regimen and vehicle via Dunnett’s test using SAS version 9.4.

## Results

### Induction of WT1-specific T cells by peptides in DSP-7888 Emulsion

**Fig. 1:**
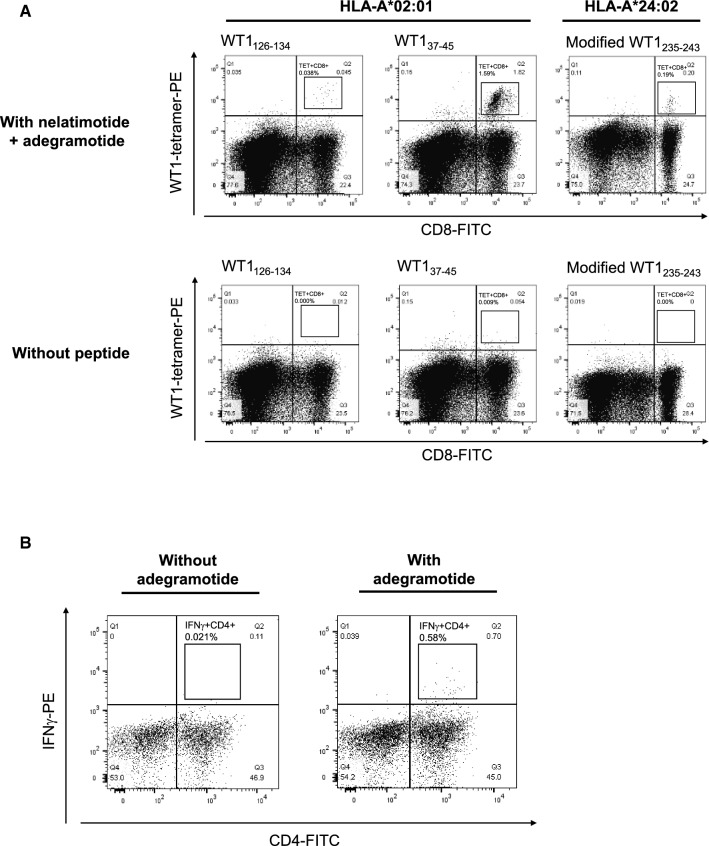
Induction of WT1-specific (A) cytotoxic T cells following in vitro exposure of human PBMCs positive for HLA-A*02:01 or HLA-A*24:02 to nelatimotide plus adegramotide or DMSO and (B) helper T cells following in vitro exposure of human PBMCs positive for HLA-DRB1*14:54/15:01, HLA-DQB1*05:03/06:03, and HLA-DPB1*04:01/04:02 to adegramotide. The presence of WT1-specific CD8^+^ T cells was detected by flow cytometry using HLA-I tetramer loaded with WT1_126–134_, WT1_37–45_, or modified WT1_235–243_. The presence of WT1-specific CD4^+^ T cells was detected by an intracellular IFN-γ stain. In Panel A, the square denotes WT1-specific CD8^+^ T cells detected via flow cytometry. In Panel B, the square denotes WT1-specific CD4^+^ T cells detected via flow cytometry. FITC, fluorescein isothiocyanate; HLA, human leukocyte antigen; IFN-γ, interferon-gamma; PBMC, peripheral blood mononuclear cell; PE, phycoerythrin; WT1, Wilms’ tumor 1

Stimulation of PBMC cultures with nelatimotide (WT1_126–134_ and modified WT1_235–243_) plus adegramotide (WT1_34–51_) led to the induction of WT1-specific CD8^+^ T cells (Fig. 1A). The CD8^+^ T cells induced by these epitopes recognized complexes of WT1 peptides and HLA-A*02:01 or HLA-A*24:02 (Fig. 1A). WT1-specific CD8 + cells were not detected in PBMCs without exposure to peptides. Treatment of human PBMCs with adegramotide led to the generation of WT1-specific CD4^+^ T cells (Fig. 1B). Collectively, these in vitro data indicate that the synthetic long peptides included in DSP-7888 Emulsion can be processed in human cells and induce both WT1-specific cytotoxic T cells and helper T cells.

**Fig. 2:**
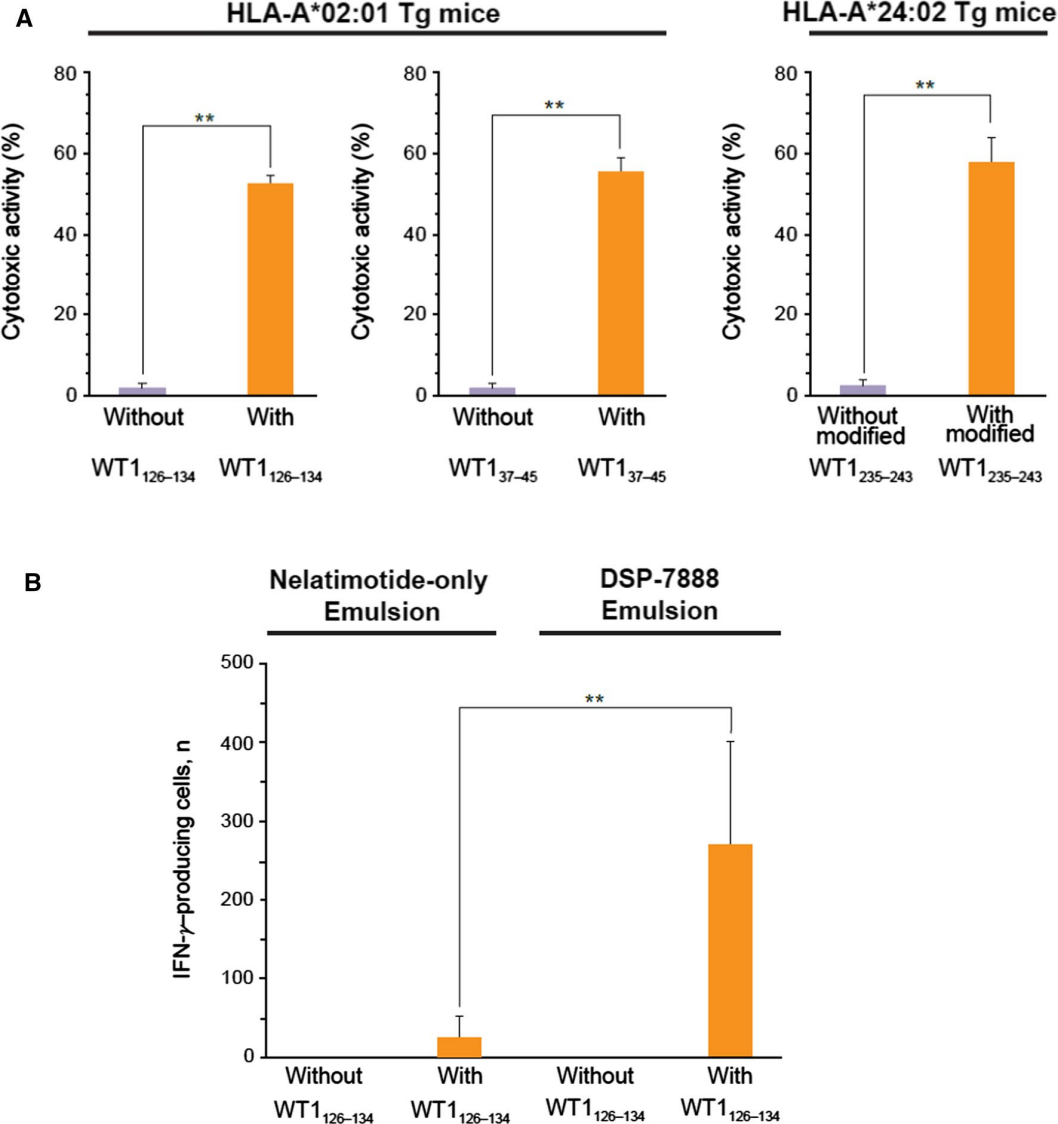
Characterization of DSP-7888 Emulsion-induced WT1-specific CD8^+^ T cells. (A) Effector T cells were harvested from the spleens of HLA-A*02:01 or HLA-A*24:02 transgenic mice administered intradermal DSP-7888 Emulsion. Cytotoxicity was measured in chromium-release assays involving mouse-derived cytotoxic T cells and HLA-I-positive target cells pulsed with or without WT1_126–134_, WT1_37–45_, or modified WT1_235–243_. Data are the mean ± standard deviation of experiments performed in triplicate. (B) Effector T cells were harvested from the spleens of HLA-A*02:01 transgenic mice administered two weekly intradermal injections of DSP-7888 or nelatimotide-only Emulsion. The number of cells specific to WT1_126–134_ was quantified using IFN-γ ELISPOT. Data are the mean ± standard deviation of measurements from five mice. **p < 0.01 (*t* test). ELISPOT, enzyme-linked immunospot; HLA, human leukocyte antigen; IFN-γ, interferon-gamma; Tg, transgenic

Next, in ^51^Cr-release assays, we evaluated the cytotoxic activity of WT1-specific CD8^+^ T cells in splenocytes derived from HLA-A*02:01 or HLA-A*24:02 transgenic mice administered intradermal DSP-7888 Emulsion or control Emulsion (vehicle solution emulsified with Montanide ISA^™^ 51 VG). In splenocytes derived from DSP-7888 Emulsion-treated HLA-A*02:01 transgenic mice, the cytotoxic activities against HLA-A*02:01–positive target cells pulsed with WT1_126–134_ or WT1_37–45_ were significantly greater than those against target cells pulsed with no peptides (*p* < 0.01) (Fig. 2A). In splenocytes derived from DSP-7888 Emulsion-treated HLA-A*24:02 transgenic mice, the cytotoxic activities against HLA-A*24:02–positive target cells pulsed with modified WT1_235–243_ were significantly greater than those against target cells pulsed with no peptides (*p* < 0.01) (Fig. 2A). As a control for the HLA restriction of the induced WT1-specific cytotoxic T lymphocytes, these experiments were also performed using target cells negative for HLA-A*02:01 or HLA-A*24:02 (as appropriate). Regardless of whether the HLA-negative target cells were cultured in the presence of splenocytes from mice administered DSP-7888 emulsion or control Emulsion, the mean cytotoxic activity in the ^51^Cr-release assays was less than 10% (data not shown). The corresponding values for splenocytes derived from mice administered control Emulsion were less than 10%, regardless of whether target cells expressed WT1 peptide/HLA complexes (data not shown). These results indicate that only CD8^+^ T cells derived from mice treated with DSP-7888 Emulsion are capable of lysing target cells presenting WT1 antigens in an HLA-restricted manner.

To assess the in vivo contributions of adegramotide to the immunotherapeutic potential of DSP-7888 Emulsion, HLA-A*02:01 transgenic mice were injected intradermally with DSP-7888 Emulsion (i.e., both nelatimotide and adegramotide) or nelatimotide-only Emulsion. Compared with mice administered nelatimotide-only Emulsion, DSP-7888 Emulsion-treated mice produced significantly greater numbers of WT1_126-134_-specific T cells (*p* < 0.01) (Fig. 2B), suggesting that adegramotide augments the ability of nelatimotide to induce WT1-specific cytotoxic T cells.

### Enhancing the antitumor potential of DSP-7888 Emulsion

**Fig. 3:**
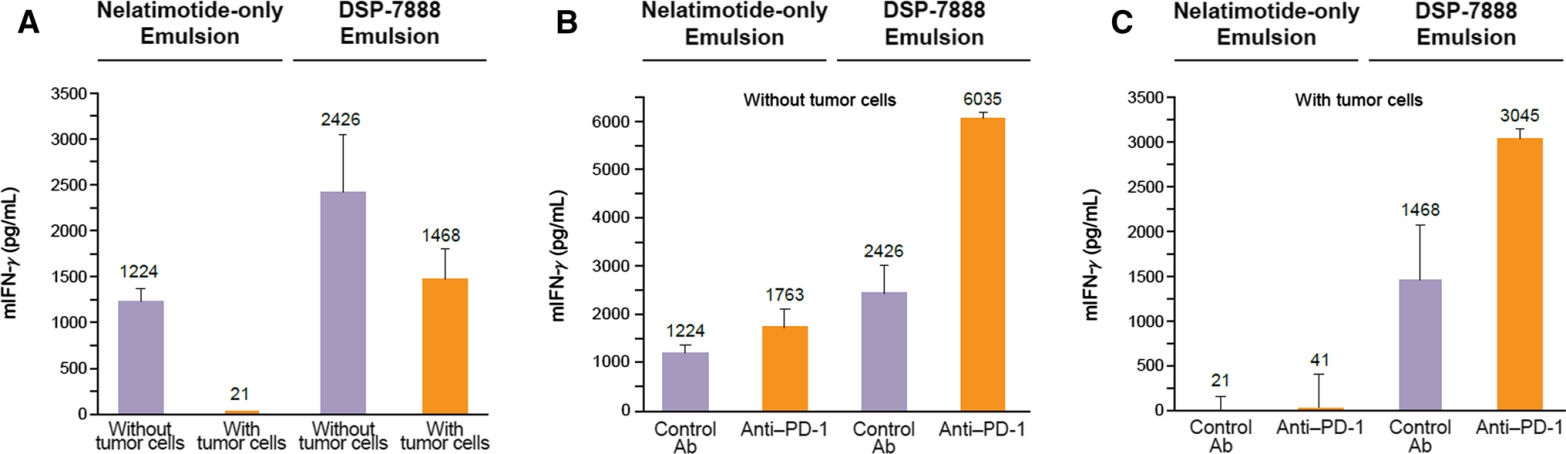
Effects of anti-PD-1 on the effector function of vaccine-induced WT1-specific CD8^+^ T cells. Splenocytes were harvested from HLA-A*02:01 transgenic mice administered DSP-7888 or nelatimotide-only Emulsion. IFN-γ secretion under antigenic stimulation was measured by ELISA in splenocytes co-cultured (A) with or without tumor cells, (B) without tumor cells in the presence of anti-PD-1 or an isotype control antibody, and (C) with tumor cells in the presence of anti-PD-1 or an isotype control antibody. Data are the mean ± standard deviation of measurements from three wells. Ab, antibody; ELISA, enzyme-linked immunosorbent assay; HLA, human leukocyte antigen; mIFN-γ, mouse interferon-gamma; PD-1, programmed cell death protein 1

Cancer cells can evade immune responses by interacting directly with host immune cells. In solid tumors, malignant cells can also influence the behavior of surrounding cells and tissues, the so-called tumor microenvironment (TME), to promote immune suppression (and, therefore, cancer growth and development). As multiple pathways contribute to the immunosuppressive nature of the TME [46], the use of combination treatment approaches is likely needed to maximize clinical outcomes. The immunogenic potential of DSP-7888 Emulsion overall, and the contributions of adegramotide in particular, when combined with a PD-1 inhibitor, were assessed using effector T cells derived from the spleens of HLA-A*02:01 transgenic mice administered DSP-7888 Emulsion or nelatimotide-only Emulsion. The isolated splenocytes were then stimulated by WT1 peptides in the presence/absence of tumor cells and incubated with either anti-PD-1 or an isotype control antibody. When splenocytes derived from HLA-A*02:01 transgenic mice treated with nelatimotide-only Emulsion were cultured in the absence of tumor cells, appreciable levels of IFN-γ were measured (Fig. 3A). However, production of IFN-γ was nearly abrogated when these splenocytes were co-cultured with irradiated LLC-HHD-WT1 tumor cells. In contrast, high levels of IFN-γ were measured in cultures of splenocytes from DSP-7888 Emulsion-treated mice, irrespective of the presence of tumor cells (Fig. 3A). Although, IFN-γ production in the tumor cell co-cultures was reduced by approximately 40% relative to the cultures lacking tumor cells.

In the cultures without tumor cells, WT1-specific CD8^+^ T cells from mice treated with nelatimotide-only Emulsion produced similar amounts of IFN-γ in the presence of anti-PD-1 or isotype control antibody (Fig. 3B). However, IFN-γ production was markedly suppressed when splenocytes from nelatimotide-only Emulsion-treated mice were co-cultured with tumor cells. In contrast, WT1-specific CD8^+^ T cells from DSP-7888 Emulsion-treated mice secreted 2.5-fold more IFN-γ when incubated with anti-PD-1 vs isotype control antibody in the absence of tumor cells (Fig. 3B). Importantly, these trends held in the tumor cell co-cultures, with the amount of IFN-γ produced by the lymphocytes from DSP-7888 Emulsion-treated mice being twofold greater in the presence of anti-PD-1 vs isotype control antibody (Fig. 3C). These data underscore the contributions of adegramotide to the effector function of nelatimotide-induced cytotoxic T cells, supporting the inclusion of both WT1-specific CD8^+^ and CD4^+^ epitopes; demonstrate how the IFN-γ–secreting ability of DSP-7888 Emulsion-induced CD8^+^ T cells is generally preserved in the presence of tumor cells; and illustrate the potential synergy between DSP-7888 Emulsion and immune checkpoint inhibition.

**Fig. 4:**
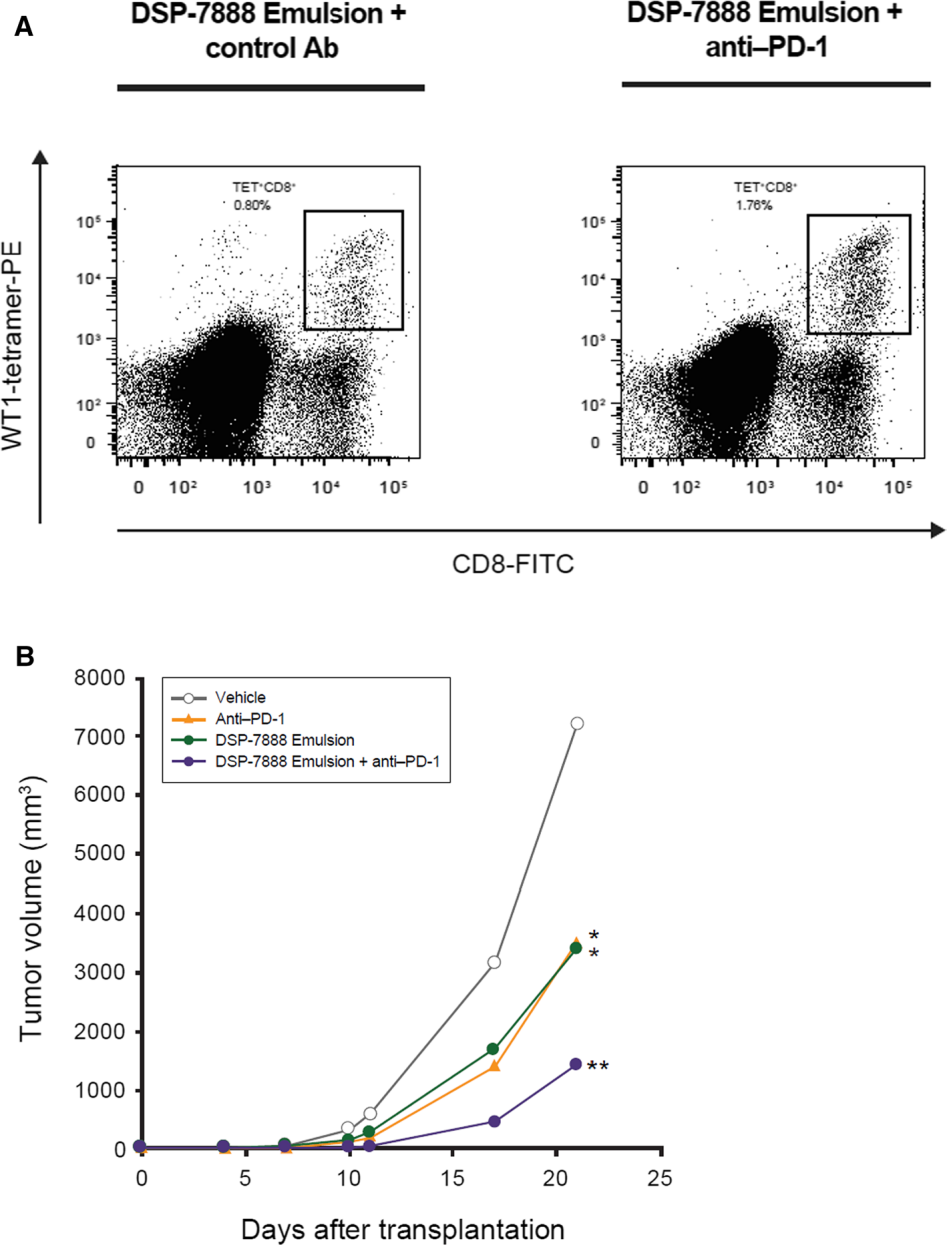
Antitumor activity of DSP-7888 Emulsion plus anti-PD-1 in HLA-A*24:02 transgenic mice implanted with WT1 antigen-positive tumors. (A) Transplanted mice were treated with DSP-7888 Emulsion plus either anti-PD-1 or an isotype control antibody. Tumors were then harvested, and the presence of WT1-specific CD8^+^ T cells was detected by flow cytometry using HLA-I tetramer. (B) Tumor size was measured in mice treated with vehicle (water emulsified with Montanide ISA™ 51 VG) plus PBS, DSP-7888 Emulsion plus an isotype control antibody, vehicle plus anti-PD-1, or DSP-7888 Emulsion plus anti-PD-1. Data are the mean ± standard deviation of measurements from five mice. *p < 0.05 compared with the vehicle-treated group (Dunnett’s test). **p < 0.01 compared with the vehicle-treated group (Dunnett’s test). Ab, antibody; CTL, cytotoxic T lymphocyte; FITC, fluorescein isothiocyanate; HLA, human leukocyte antigen; PD-1, programmed cell death protein 1; PE, phycoerythrin

The antitumor activity of combination treatment with DSP-7888 Emulsion and anti-PD-1 was explored in HLA-A*24:02 transgenic mice implanted with WT1 antigen-positive tumors. The number of tumor-infiltrating WT1-specific CD8^+^ lymphocytes was demonstrated to be two-fold higher in mice treated with DSP-7888 Emulsion plus anti-PD-1 vs DSP-7888 Emulsion plus an isotype control antibody (1.76 vs 0.80%) (Fig. 4A). This finding is supported by measured changes in tumor volume. Mice administered DSP-7888 Emulsion plus anti–PD-1 experienced significantly greater reductions in tumor size than mice treated with vehicle plus PBS or with either agent alone (Fig. 4B).

## Discussion

**Fig. 5:**
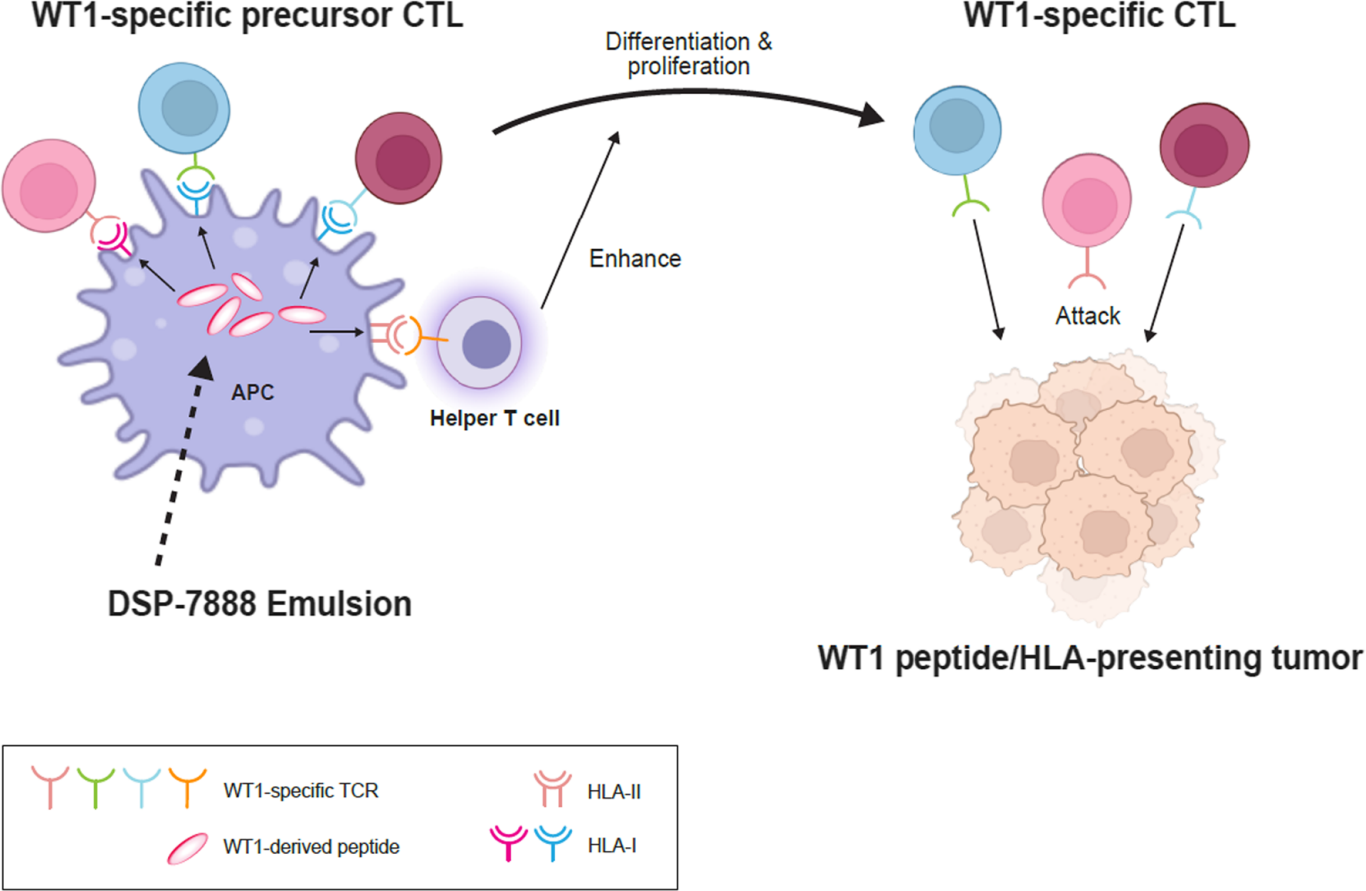
Mechanism of action DSP-7888 Emulsion. CTL, cytotoxic T lymphocyte; HLA, human leukocyte antigen; TCR, T cell receptor; WT1, Wilms' tumor 1

Through a series of in vitro and in vivo preclinical experiments, we have shown that DSP-7888 Emulsion, a WT1-based therapeutic cancer vaccine, can induce HLA-restricted WT1-specific cytotoxic T cells and helper T cells. The ability of DSP-7888 Emulsion to activate both types of effector T cells is attributable to its component peptides: nelatimotide, which is composed of two WT1-specific CD8^+^ T cell epitopes, and adegramotide, a WT1-specific CD4^+^ T cell epitope with an embedded CD8^+^ T cell epitope. Once taken up by antigen-presenting cells at the vaccination site, nelatimotide and adegramotide are thought to be carried to nearby lymph nodes and processed for presentation on HLA molecules. WT1-specific CD8^+^ and CD4^+^ T cells activated by the HLA-antigen complex then migrate to the TME, where they attack WT1-positive cancer cells and exhibit an antitumor effect (Fig. 5). In completed phase 1/2 study of DSP-7888 in patients with higher-risk myelodysplastic syndrome (MDS) who had failed prior azacytidine treatment, intradermal DSP-7888 Emulsion treatment was well tolerated and a total of 78.7% of patients experienced a positive WT1-specific immune reaction (IR) defined as delayed type hypersensitivity reaction-positive or WT1-specific CD8^+^ T cell positivity [27]. Compared with patients without a WT1-specific IR, those with a positive WT1-specific IR experienced significantly longer overall survival. These findings suggest that nelatimotide and adegramotide work in patients with MDS as we expected. Montanide ISA™ 51 VG, the adjuvant in DSP-7888 Emulsion, increases immunogenicity by stabilizing vaccine peptides and slowing their systemic release, allowing time for dendritic cells and macrophages to arrive at the injection site and absorb antigen. However, it may have the unintended effect of trapping cytotoxic T cells at the vaccination site, thereby suppressing their antitumor potential [47]. Such negative effects have been shown to be mitigated by the delivery of longer (20 amino acids) vs shorter (9 amino acids) peptide sequences [47]. DSP-7888 Emulsion contains nelatimotide with 19 amino acids and adegramotide with 18 amino acids, rather than shorter peptides, which may minimize the capture of WT1-specific CD8^+^ T cells at the vaccination sites in clinical settings.

Our studies also demonstrated the contributions of adegramotide to augmenting CD8^+^ T-cell numbers and how the cytotoxic T lymphocytes induced by nelatimotide and adegramotide are capable of functioning within the immunosuppressive TME. Regarding this last point, we discovered that the WT1-specific CD8^+^ T cells induced by nelatimotide-only Emulsion (i.e., DSP-7888 Emulsion without adegramotide) did not become activated when co-cultured with tumor cells, including when treated with anti-PD-1, but DSP-7888 Emulsion-induced CD8^+^ T cells did. This suggests that cytotoxic T lymphocytes alone are insufficient for robust anti-cancer activity, which may explain the limitations of WT1 peptide-based therapeutic cancer vaccine containing only CD8^+^ T-cell epitopes.

Based on this study illustrating the ability of anti-PD-1 and DSP-7888 Emulsion to enhance each other’s antitumor effects, there is potential for DSP-7888 Emulsion to be used in combination with immune checkpoint inhibitors in the clinical setting. Considering that cancer vaccines containing neoantigen-derived peptides lead to an accumulation of the number of cancer antigen-specific CTLs in GBM tumor microenvironment [48], PD-1 inhibition may be effective in tumor types with few tumor-infiltrating T cells by combined with agents that strongly induce antitumor T cell immunity, such as DSP-7888 Emulsion. Interestingly, PD-1 inhibition has potential to restore CTL induction by a cancer vaccine [49], suggesting that the combination of cancer vaccines and anti-PD-1 may cause long-term tumor remission in patients. Clinical trials of DSP-7888 Emulsion in combination with anti-PD-1 (nivolumab or pembrolizumab) are underway. Results from the dose-finding part of a Phase 1b/2 study of DSP-7888 Emulsion when administered in combination with an anti-PD-1 for the treatment of advanced solid tumors (NCT03311334) have demonstrated preliminary antitumor activity of DSP-7888 Emulsion in combination with anti-PD-1 in two patients who progressed on prior immune checkpoint inhibitors treatment [50]. Further investigation is also warranted, including the combination of DSP-7888 Emulsion with a wide range of treatments other than PD-1 inhibition.

## Data Availability

The data that support the findings of this study are available from the corresponding author, MG, upon reasonable request.

## Abbreviations

CTL: Cytotoxic T lymphocyte
DMSO: Dimethylsulfoxide
ELISPOT: Mouse IFN-γ Enzyme-Linked Immunospot
FACS: Fluorescence-activated single cell sorting
FITC: Fluorescein isothiocyanate
GBM: Glioblastoma multiforme
HLA: Human leukocyte antigen
HTL: Helper T lymphocyte
IFN-γ: Interferon-gamma
IR: Immune reaction
MDS: Myelodysplastic syndrome
PBMC: Peripheral blood mononuclear cell
PD-1: Programmed cell death protein 1
PD-L1: Programmed cell death-ligand 1
TME: Tumor microenvironment
WT1: Wilms’ tumor 1
